# Interplay of the Quality of Ciprofloxacin and Antibiotic Resistance in Developing Countries

**DOI:** 10.3389/fphar.2017.00546

**Published:** 2017-08-21

**Authors:** Deepali Sharma, Rahul P. Patel, Syed Tabish R. Zaidi, Md. Moklesur Rahman Sarker, Qi Ying Lean, Long C. Ming

**Affiliations:** ^1^Pharmacy, School of Medicine, University of Tasmania, Hobart TAS, Australia; ^2^Department of Pharmacy, State University of Bangladesh Dhaka, Bangladesh; ^3^Vector borne Diseases Research Group, Pharmaceutical and Life Sciences CoRe, Universiti Teknologi MARA Shah Alam, Malaysia; ^4^Faculty of Pharmacy, Universiti Teknologi MARA Bertam, Malaysia; ^5^School of Pharmacy, KPJ Healthcare University College Negeri Sembilan, Malaysia

**Keywords:** antibiotic resistance, ciprofloxacin, quality assurance, substandard drug, spurious drug, pharmacovigilence and drug monitoring, drug regulatory system, fluoroquinolones

## Abstract

Ciprofloxacin, a second generation broad spectrum fluoroquinolone, is active against both Gram-positive and Gram-negative bacteria. Ciprofloxacin has a high oral bioavailability and a large volume of distribution. It is used for the treatment of a wide range of infections including urinary tract infections caused by susceptible bacteria. However, the availability and use of substandard and spurious quality of oral ciprofloxacin formulations in the developing countries has been thought to have contributed toward increased risk of treatment failure and bacterial resistance. Therefore, quality control and bioequivalence studies of the commercially available oral ciprofloxacin formulations should be monitored. Appropriate actions should be taken against offending manufacturers in order to prevent the sale of substandard and spurious quality of ciprofloxacin formulations.

## Introduction

Antibiotics, from natural or synthetic sources, inhibit the growth of microorganisms or kill the bacteria (Barbereau, 2006; Nayyar et al., 2015; Reis et al., 2016). It is now evident that falsified and counterfeit medications can cause treatment failure and antibiotic resistance (World Health Organisation, 2016). According to the European Commission, falsified or fake medications are the ones that pass themselves off as real, authorized medications without being evaluated for their quality, safety and efficacy (European Commission, 2016). Such medications may contain substandard active ingredients, which are of low quality and/or have incorrect amount, either too high or too low (Lee et al., 2017). Counterfeit medications are, terminologically different from falsified medications. Counterfeit refers to medications that do not comply with regulation on intellectual and industrial property rights, for example, unregistered medicines sourced from parallel import (Fadlallah et al., 2016; European Medicines Agency, 2017). In developing countries including India, Pakistan, Bangladesh, Ethiopia, Nigeria, Kenya, Uganda, and Nigeria the control and monitoring of drug manufacturing, marketing, distribution, and consumption is not strictly controlled resulting in the availability of falsified medications. According to World Health Organisation, up to 10% of medications may be falsified worldwide. Of these, 50% are estimated to be antimicrobial agents of which 78% are from Asian and African countries (World Health Organisation, 2016). Among falsified antibiotics, 43% have no active ingredients, 24% are of bad quality, 21% are with less quantity of an active ingredient, and 7% are with wrong ingredients (Delepierre et al., 2012; Kratochwill et al., 2015). It was also found that the falsified antibiotics consisted of 50% of beta lactams, 12% quinolones, 11% macrolides, 7% cyclins, and 20% other antibiotics. India is reported as the leading country with the highest number of falsified antibiotics followed by Burma and Nigeria (Kelesidis and Falagas, 2015). More than 60,000 pharmaceutical formulations produced in India are not registered (Bhargava and Kalantri, 2013) and the availability of substandard antibiotics is increasing worldwide. This review mainly focuses on the availability of substandard formulations of generic ciprofloxacin, a type of quinolone, and its impact on bacterial resistance and treatment failure.

### Quinolones

**FIGURE 1:**
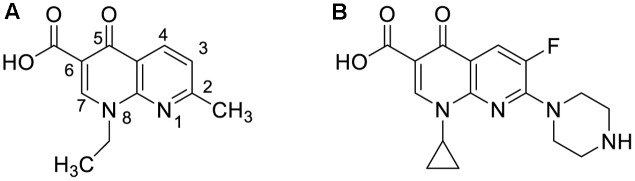
Chemical structures of nalidixic acid **(A)** and ciprofloxacin **(B)**.

Quinolones are classified according to their generations (Drlica, 1999). Nalidixic acid, the first generation quinolone, is effective against Gram-negative bacteria excluding *Pseudomonas* species (von Rosenstiel and Adam, 1994). The structure of nalidixic acid is shown in **Figure 1A**. After intravenous administration, it is eliminated very rapidly and therefore sufficient systemic levels required for antibacterial effect cannot be achieved. Its plasma half-life is about one and half hour, and because of its rapid renal elimination, its use for the treatment of systemic infections was not possible (von Rosenstiel and Adam, 1994). However, it acted as an ideal urinary antiseptic, and, therefore, was used for the treatment of urinary tract infections. Emergence of bacterial resistance and high incidence of neurological, dermatological and gastrointestinal side effects eventually limited its use in clinical practice. Norfloxacin, ciprofloxacin, ofloxacin, enoxacin, and lomefloxacin are examples of second generation quinolones. Third generation quinolones include moxifloxacin, levofloxacin, gatifloxacin, and sparfloxacin. Trovafloxacin is an example of fourth generation quinolone (Neu, 1989).

Quinolones exert the potent antibacterial effect by binding to bacterial enzymes, DNA gyrase and topoisomerase IV. This binding results in the formation of quinolone–enzyme–DNA complex. Shortly after binding, the enzyme undergoes conformational changes. The enzyme breaks the DNA and the drug prevents religation of the broken DNA strands, thus preventing DNA replication. Ultimately, this results in the damage to bacterial DNA and thus cell death (Stefan et al., 2016). Unlike first generation, second generation quinolones are effective against *Pseudomonas* species, some Gram-positive bacteria (including *Staphylococcus aureus* but not *Streptococcus pneumoniae*) and some atypical pathogens. The antimicrobial spectrum of third generation quinolones is similar to that of second generation but they have extended Gram-positive and atypical microorganisms. Fourth generation quinolones are as effective as third generation quinolones against Gram-negative, Gram-positive and atypical pathogens but have broad anaerobic coverage (Symonds and Nix, 1992).

Quinolones have a good oral bioavailability (Drlica, 1999). Unlike most classes of antibiotics, the bioavailability of oral quinolones (except norfloxacin) is comparable to quinolones administered intravenously (Just, 1993; von Rosenstiel and Adam, 1994). Therefore, often dose adjustment is not necessary while switching from intravenous to oral quinolones. The extent of absorption of orally administered quinolones is significantly decreased if co-administered with products containing positively charged cations such as zinc, aluminum, calcium, and magnesium. The positively charged cations form insoluble drug-cation complexes in the gastrointestinal tract (GIT). Quinolones have a large volume of distribution and their distribution in urinary tract and respiratory tissues is of important because they are effective against microorganisms commonly responsible for urinary tract and respiratory tract infections (Davis et al., 1996).

### Physicochemical and Pharmacokinetics Characteristic of Ciprofloxacin

Ciprofloxacin, a second generation quinolone, has a pH dependent solubility (Jaman et al., 2015). It’s solubility is maximum at pH below 5 and minimum near to the isoelectric point (pH 7) (Torniainen et al., 1996; Oishi et al., 2011). Besides, ciprofloxacin in zwitterionic form was found to be most sensitive to photodegradation at slightly basic pH (Torniainen et al., 1996). For example, at pH 8.6, the percentage of ciprofloxacin photodegradation is reported to be 85%. On the other hand, the percentage of ciprofloxacin photodegradation decreases to 15% at pH 3.0 and pH 4.0 whereas the extent of photodegradation is similar at pH 6.0 and pH 10.6.

Ciprofloxacin is structurally similar to nalidixic acid (**Figure 1B**) (Davis et al., 1996). The sixth position controls the mechanism of action and bacterial potency of quinolones. Unlike nalidixic acid, ciprofloxacin contains the fluorine atom at position number 6. Due to this single change, ciprofloxacin is approximately 100 times more potent than nalidixic acid. Positions 1 and 7 seven control the potency spectrum and pharmacokinetics of quinolones. *N*-ethyl and methyl groups present on the first and seventh position of nalidixic acid are replaced with a six-membered ring and *N*-cyclopropane respectively in ciprofloxacin resulting in ciprofloxacin’s extended Gram-negative activity, higher potency, better tissue penetration, greater bioavailability, and longer plasma half-life (Sanchez et al., 1992; Tillotson, 1996). It is used for the treatment of a wide range of infections, such as complicated urinary tract infections, bone or joint infections, cystic fibrosis, prostatitis, typhoid, shigellosis, chronic suppurative otitis media, and epididymo-orchitis caused by susceptible bacteria. Common adverse drug reactions of ciprofloxacin include nausea, vomiting, diarrhea, and abdominal pain. Thrombophlebitis at intravenous infusion site, phototoxicity, transient hearing impairment, and crystalluria are some of the infrequent and rare reported adverse drug reactions of ciprofloxacin (Davis et al., 1996).

Following oral administration, ciprofloxacin is rapidly absorbed in the GIT by passive diffusion and reaches the peak serum concentration within 2 h. The absorption rate is affected by intestinal pH, with greater absorption in the duodenum and proximal jejunum than in the distal small intestine (Campoli-Richards et al., 1988; Davis et al., 1996). Ciprofloxacin demonstrates a concentration-dependent permeability and it is widely distributed in body tissues and fluids including bile, prostatic tissues, gingival fluid, and lungs. Clearance of ciprofloxacin is by both renal and non-renal pathways (Micromedex, 2016). Approximately, two third of metabolites are excreted in the urine and 15% in feces (Olivera et al., 2011).

## Bioavailability and Bioequivalence of Innovator and Generic Ciprofloxacin

Generic equivalents of brand-name drugs (innovator drugs) have been introduced in the global healthcare market to lower the cost of medications (Kaushal et al., 2016). A generic drug product is assumed to be bioequivalent to the brand-name drug product (innovator drug serve as the reference) if there is no statistically significant difference in the rate and extent of absorption of the active ingredient when administered at similar dose (Turner, 2016). Comparative bioavailability studies need to be established before generic drug formulations are commercially available to consumers. This is to assure that the safety and efficacy of generic drug formulations are comparable to the corresponding innovator drugs (Oishi et al., 2011).

Several human studies comparing the bioequivalence of generic ciprofloxacin formulations to brand-name formulations have been reported before. The reported studies compared various pharmacokinetic parameters, such as extent of absorption [area under the curve (AUC)], maximum plasma concentration (C_max_), time to reach C_max_ (T_max_) and half-life, between generic and respective reference formulations. At three different oral doses (250, 500, or 750 mg), pharmacokinetic parameters of generic formulations were equivalent to the reference formulations. As expected, a high dose of ciprofloxacin (750 mg), often used in severe and/or complicated infections, showed higher C_max_ than 500 and 250 mg dose of ciprofloxacin. T_max_ and half-live values after the administration of 750 and 250 mg varied from 1.38 to 0.69 h and 4 to 5 h, respectively (Plaisance et al., 1987). The oral bioequivalence of several ciprofloxacin formulations at different doses are presented in **Table 1** (Azad et al., 2007; Hassan et al., 2007; Khan et al., 2009; Fahmy and Abu-Gharbieh, 2014). These studies reported that the rate and extent of absorption of the tested generic formulations are similar to those observed with brand-name formulations and therefore these generic and brand-name formulations could be used interchangeably (Azad et al., 2007; Valizadeh et al., 2012). However, 100s of ciprofloxacin generic formulations of ciprofloxacin are widely available worldwide. For example, there are more than 75 pharmaceutical companies in India that manufacture generic ciprofloxacin. Therefore, studies comparing the bioequivalence of such formulations to brand-name formulations are warranted.

**Table 1:** Mean (range) pharmacokinetic parameters from a single dose of reference and test tablets of ciprofloxacin.

Name and	Number of healthy		AUC∞				
strength	volunteers	Type	(mg/L^∗^h)	Cmax (mg/L)	Half-life (h)	Tmax (h)	Reference
Ciproxin^®^ (Bayer health care pharmaceuticals); 250 mg	24 adults	Reference formulation	6.92 (6.18–8.49)	1.46 (141–1.50)	4.52 (3.79–5.52)	1.20 (1.0–1.50)	Azad et al., 2007
Quinox^®^ (Eskayef Bangladesh, Ltd.); 250 mg		Test formulation	6.67 (6.18–8.48)	1.49 (1.39–1.62)	4.33 (3.90–6.46)	1.00 (1.0–1.50)	
Ciproxin^®^ (Bayer Laboratory); 500 mg	18 adults	Reference formulation	11.95 (7.96–19.51)	2.16 (1.19–3.23)	–	1.56 (1.0–2.5)	Morera et al., 2001
Cinaflox^®^ (Stein laboratory); 500 mg		Test formulation	11.15 (7.77–16.05)	1.99 (1.41–2.79)	–	1.63 (1.0–2.5)	
Ciprobay^®^ (Bayer Vital GmbH and Co., Germany); 750 mg	24 adults	Reference formulation	15.70 (8.92–24.55)	3.32 (1.91–5.18)	5.00 (3.88–6.43)	1.49 (0.75–4)	Cuadrado et al., 2004
Brand not disclosed (Dr. August Wolff GmbH and Co., Germany); 750 mg		Test formulation	15.31 (8.92–22.51)	3.50 (2.12–5.15)	5.06 (3.95–6.31)	1.38 (0.75–2.5)	


### Bacterial Resistance to Ciprofloxacin

Fluoroquinolone resistance occurs quickly and is related to molecular evolutionary biology and direct response toward drug pressure (Redgrave et al., 2014; Fasugba et al., 2015). The most common type of clinically significant resistance is alterations in the quinolone enzymatic targets, which is caused by specific mutation of gyrase and topoisomerase IV which in turn weakens the interactions between quinolones and bacterial enzymes (Aldred et al., 2014). Plasmid mediated resistance is another mechanism by which bacteria can acquire resistance to quinolones. It is proposed that bacteria acquire plasmid genes that code for proteins responsible for protecting bacterial enzymes, DNA gyrase and topoisomerase IV, from the effect of quinolones (van Hoek et al., 2011; Jacoby et al., 2014). Chromosome mediated resistance is developed due to under expression of porins or ever expression of cellular efflux pumps decreasing the cellular concentration of quinolones (Naeem et al., 2016; Turner, 2016).

Ciprofloxacin has been shown to be active against isolates of various Gram-positive and Gram-negative bacteria, both *in vitro* and *in vivo* (Tamma et al., 2012). It is one of the antibiotics used for respiratory, urinary tract, intestinal and abdominal infections caused by various pathogens including *Escherichia coli, Haemophilus influenzae*, other *H.* spp*., Neisseria gonorrhoeae, N. meningitides, Moraxella catarrhalis, Klebsiella pneumoniae, Legionella pneumophila, Moraxella catarrhalis, Proteus mirabilis*, and *Pseudomonas aeruginosa*, methicillin-susceptible *Staphylococcus aureus, Streptococcus pneumoniae, Staphylococcus epidermidis, Enterococcus faecalis*, and *Streptococcus pyogenes* (Rodriguez et al., 2009, 2015; Vesga et al., 2010; Agudelo and Vesga, 2012; Tamma et al., 2012). Nonetheless, concerns in relation to the appropriate use of fluoroquinolones and quality of their pharmaceutically equivalent formulations have been raised after the reported therapeutic failures of fluoroquinolones including ciprofloxacin when used for the treatment of various infections (Bowen et al., 2015; De Lappe et al., 2015; Kim et al., 2015). One of the main reasons for the reported treatment failure is thought to be due to increased bacterial resistance to fluoroquinolones (Jabeen et al., 2015).

Bacterial resistance to ciprofloxacin has been reported in the early 90s and is continuously rising ever since (Cahana et al., 1995; Corti et al., 1995; Hakanen et al., 1999). For example, a study presented prevalence of ciprofloxacin among 480 isolates obtained from patients with urinary tract infections. The resistance rate to ciprofloxacin was 15.0%. High resistance to ciprofloxacin was detected among *Acinetobacter haemolyticus* (28.6%), *Staphylococcus saprophyticus* (25.0%) (El Astal, 2005). Relatively recent systematic review and meta-analysis of observational studies concluded that the resistance rate of urinary *E. coli* to ciprofloxacin is increasing and the policy on appropriate use of ciprofloxacin should be developed and enforced especially in developing countries (Fasugba et al., 2015). Also, a study conducted in Brazil reported a much higher than expected rate of bacterial resistance to ciprofloxacin (Reis et al., 2016). Ciprofloxacin resistance varies significantly from country to country with the highest resistance reported in developing countries (Fasugba et al., 2015). Due to an increase in resistance, Infectious Diseases Society of America (IDSA) recommends to limit the use of fluoroquinolones to infections, where other antibiotics cannot be used due to reasons including associated side effects or causative organisms are found to be resistance to alternative antibiotics.

### Potential Reasons for Increased Bacterial Resistance to Ciprofloxacin

There could be a number of reasons behind the observed increase in bacterial resistance to ciprofloxacin. One reason could be the overuse or misuse of ciprofloxacin likely to be due to wide spread availability of generic versions of ciprofloxacin. Overuse or misuse of antibiotics is known to promote bacterial resistance and is likely to limit the effectiveness of ciprofloxaxin. After the introduction of generic ciprofloxacin in the market, an increase in consumption of ciprofloxacin was observed. A study in Denmark showed that total consumption of oral ciprofloxacin increased significantly from 0.13 to 0.33 defined daily dose/1000 inhabitant-days. At the same time, the rate of ciprofloxacin resistance has increased by 200% (Jensen et al., 2010). In another study, it was reported that the resistance of isolated *E. coli* obtained from patients with UTI increased proportionally with the use of quinolones (Fasugba et al., 2015). Similarly, a wide spread use of ciprofloxacin was thought to be responsible for a significant rise in ciprofloxacin resistance over time (Fasugba et al., 2015). As the driving force for the prevalence of antibiotic resistance is the extent of drug use, there is a positive correlation between consumptions of quinolones and antibiotic resistance (Jensen et al., 2010). In certain parts of the densely populated developing countries such as Brazil, Indonesia, Pakistan, India and China, there are hot spots for emergence and spread of antibiotic resistance (Huynh et al., 2015). It was speculated that South Asia is the reservoir for the global spread of ciprofloxacin-resistant infections caused by various types of bacteria including *S. saprophyticus, P. aeruginosa, K. pneumoniae*, multidrug-resistant *Salmonella enterica* serotype *Typhi/Paratyphi*, *E. coli*, and *Shigella sonnei* (Rabaa et al., 2016). Mulder et al. (2016) reported that the use of two, three, or more prescriptions of fluoroquinolones during a single infection was associated with bacterial resistance to ciprofloxacin. Moreover, in certain parts of Africa, Asia and Middle East, the use of prescription drugs without medical advice is reported to be 100, 58, and 39%, respectively. The frequent use of prescription drugs in such communities has contributed to increase bacterial resistance (Morgan et al., 2011).

Another reason for the observed increase in bacterial resistance to ciprofloxacin could be due to easy accessibility to substandard and spurious formulations of ciprofloxacin. Substandard and spurious generic versions of drugs are therapeutically ineffective when used clinically (Agudelo and Vesga, 2012). As a consequence, treatment fails and this potentially enhances the selection of bacterial resistance (Rodriguez et al., 2009). Weir et al. (2005) collected 130 ciprofloxacin eye drop samples sold in India and then randomly selected 30 samples for the analysis of ciprofloxacin concentration. The authors reported that in 20% samples, the concentration of ciprofloxacin was below the standard recommended range. In addition, a number of formulations had the concentration of ciprofloxacin sufficiently lower to have negative impact on clinical outcome leading to increased risk of bacterial resistance. Prazuck et al. (2002) collected three generic oral formulations of ciprofloxacin manufactured in India. The content of ciprofloxacin in two out of three formulations varied by more than -20%. One collected formulation in fact expired 11 months ago. This study highlighted the availability of poor quality of ciprofloxacin formulations in India. Previous studies have also shown that the production of substandard drugs is common in Southeast Asia (Newton et al., 2001). Bate et al. (2009) determined the quality of various antibiotics, including ciprofloxacin, commercially available in two major Indian cities; namely Chennai and Delhi. They collected 50 and 53 ciprofloxacin treatment packs from Delhi and Chennai pharmacies respectively. The quality was determined based on the content of ciprofloxacin and disintegration test. Approximately, 10% and 6% of ciprofloxacin collected from Delhi and Chennai, respectively failed either one or two quality tests. There could be a number of reasons behind the availability of substandard ciprofloxacin worldwide. However, one of the common reasons could be the lack of regulatory policies in relation to both appropriate manufacturing practice and quality assurance procedures.

### Way Forward

Currently, approaches to determine bioavailability and bioequivalence of pharmaceutical products has been largely standardized. In United States, the sale of generic drugs is approved by Food and Drug Administration when they meet all the regulatory requirements provided in the Code of Federal Regulations (Kaushal et al., 2016). The regulatory environment of the country of marketing is important to assure the assessment of bioequivalence of drug products. WHO has made tremendous progress in developing international consensus for standardizing and harmonizing the regulatory requirement, mainly for manufacturing control, safety and efficacy of new drugs and assessing bioequivalence of generics. The national, regional organization and regulatory authorities of developing countries including Central Drugs Standard Control Organization (CDSCO), South African Development Community (SADC), and Association of South East Nations (ASEAN) should strive to provide comprehensive regulatory guidelines related to product quality testing and conduct of bioequivalence studies (Midha and McKay, 2009). Different resources, expertise and stringent regulation and enforcement are required to ensure proper implementation to ensure generic formulations are safe and effective (Sarker et al., 2016). Moreover, post-marketing surveillance is also necessary as the use of higher strength drug than the labeled value can result in toxic effects due to overdose whereas taking substandard agents can result in treatment failure. Similarly, strict monitoring of the production of ciprofloxacin formulations by drug regulatory agencies as well as post-marketing quality control studies are required to prevent and identify the substandard and spurious brands of oral ciprofloxacin in order to prevent the risk of treatment failure and antibiotic resistance.

## Conclusion

The substandard quality ciprofloxacin is potentially the driving force for ciprofloxacin resistance. The availability and use of substandard quality ciprofloxacin would jeorpadise the clincial efficacy of this broad spectrum antibitoic. Drug quality assurance and strict laws and regulations for manufacturing, importation, distribution, and sale of ciprofloxacin are required to prevent the availability and use of substandard ciprofloxacin formulations, which in turn will minimize the risk of treatment failure and antibiotic resistance. An international comprehensive policy for addressing the resistance of ciprofloxacin and other fluroquinonoles in general is much needed.

## Author Contributions

RP, SZ, and LM conceived the concept; DS, QL, RP, SZ, MS, and LM wrote the initial draft; DS, RP, SZ, MS, QL, and LM finalized the manuscript. All authors contributed toward revising the paper and agreed to be accountable for all aspects of the work.

## Conflict of Interest Statement

The authors declare that the research was conducted in the absence of any commercial or financial relationships that could be construed as a potential conflict of interest.
